# Repurposing clofazimine as an antibiotic to treat cholera: Identification of cellular and structural targets

**DOI:** 10.1016/j.jbc.2025.110458

**Published:** 2025-07-04

**Authors:** Ming Yuan, Martín Andrés González Montalvo, Yuyao Hu, Karina Tuz, Oscar X. Juárez

**Affiliations:** Department of Biological Sciences, Illinois Institute of Technology, Chicago, Illinois, USA

**Keywords:** *Vibrio cholerae*, NQR, antibiotic resistance, clofazimine, Lamprene, drug repurposing

## Abstract

*Vibrio cholerae* has shaped the face of human civilization through at least seven pandemic waves. The current wave shows multidrug resistance, has produced enormous human and economic losses, as well as humanitarian crises, and has the potential to collapse the health care system of entire countries. Antibiotic resistance in this and other pathogens is an urgent threat that remains unaddressed because of the significant costs to develop new antibiotics. In this work, we have tested several Food and Drug Administration–approved phenazines and phenothiazines and have identified that clofazimine (Lamprene) shows strong antibiotic effects against *V*. *cholerae* cells in culture and in an *in vitro* infection model, at concentrations well below the clinically used doses in humans. Our results show that in an animal model, clofazimine is as effective as ampicillin in the treatment of cholera. In addition, clofazimine shows strong antivirulence properties, almost completely inhibiting cholera toxin production. The characterization of *V*. *cholerae* metabolism allowed us to identify that clofazimine’s main target in this pathogen is the respiratory complex NQR, an essential enzyme that plays a crucial role in energy metabolism, virulence factor production, and multidrug resistance, which is widely distributed among pathogenic bacteria. Biochemical and computational analyses show that the structural target of clofazimine is the catalytically active ubiquinone-binding site, which is a unique structural motif, not found in any human protein, making it an ideal pharmacologic target. These results show that clofazimine can be repurposed to treat cholera and open opportunities to develop a novel class of antibiotics that target NQR.

Over the last 20 years, seven *Vibrio cholerae* pandemics have occurred, causing millions of deaths at a global scale (1). In 2019, the World Health Organization reported close to 1 million cases of cholera worldwide (https://reliefweb.int/report/world/weekly-epidemiological-record-wer-11-september-2020-vol-95-no-37-pp-441-448-enfr). However, it is estimated that the actual number of cases could be up to 4 million annually, with around 150,000 deaths (2). Tetracycline, fluoroquinolones, and azithromycin are the mainstay antibiotics used in the treatment of cholera. Unfortunately, the emergence of antibiotic-resistant strains has increased treatment failure (3, 4). A recent study indicates that multidrug-resistant *V*. *cholerae* O1 is highly prevalent worldwide, producing 35% of all diarrhea cases in older children and adults (5). In addition, other alarming trends have become evident: two studies in Nepal and a five-country study in sub-Saharan Africa show that 100% of cholera cases were multidrug resistant (6, 7, 8). In India, more than 400 clinical *V*. *cholerae* strains have been identified showing antibiotic resistance (9). A recent example that illustrates the risks associated with this pathogen is the 2010 outbreak in Haiti, after a devastating earthquake that caused the death and displacement of millions of people. This outbreak is attributed to a South Asian multidrug-resistant strain introduced through human activity, which contaminated the Meye Tributary System of the Artibonite River (10). This cholera outbreak produced more than 800,000 cases, 10,000 deaths, and collapsed the health care system in the country. The emergence of novel diseases, alongside the persistent challenge of cholera, has the potential to severely compromise or collapse the health care system in many other countries. In addition, environmental factors increase the risks associated with cholera’s spread and expansion, such as seasonal high temperatures, which have increased the number of cholera cases in East Africa, Zambia, South China, and Iran in recent years (1, 11, 12, 13, 14). Moreover, climate change models predict that *V*. *cholerae* will spread its range dramatically in the next 30 years, affecting regions that have remained untouched until now (11). New treatments are desperately needed to treat cholera in developing countries, to prevent humanitarian crises, and to protect military and health care personnel deployed to conflict areas around the world where the disease is prevalent.

Due to the over 25-year gap in the development of new antibiotics and to the close to 2-billion-dollar investment to develop new therapeutics, there have been considerable efforts to repurpose Food and Drug Administration (FDA)–approved drugs as antibiotics. Over the last decade, the antibiotic activity of phenothiazines and phenazines (Phes) has started to be re-evaluated (15, 16, 17). Phenothiazines and Phes are tricyclic compounds that contain nitrogen and/or sulfur in positions 5 and 10 of the ring (Fig. 1*A*). Phenothiazines have been used in the clinic as neuroleptics with antipsychotic properties (18) and can also be used in the treatment of Alzheimer’s disease (19, 20). Interestingly, early studies showed that phenothiazines also have antimicrobial activities (17). On the other hand, Phes have been used in the treatment of leprosy and tuberculosis (21). These molecules represent a viable option to address antibiotic resistance in pathogenic bacteria, especially since they have FDA clearance and a well-documented safety profile. In this work, we are reporting that among several FDA-approved phenothiazines and Phes, clofazimine (Lamprene), an inexpensive orphan drug (22), can be repurposed to treat cholera. Our results show that clofazimine has potent antibiotic properties against laboratory and clinical *V*. *cholerae* strains in culture and in an *in vitro* infection model of human intestinal epithelial cells, with MIC_50_ in the low micromolar range, well below the clinical doses used in humans. In addition, our data show that clofazimine, at a concentration used to treat tuberculosis, is as effective as ampicillin in a *V*. *cholerae* animal infection model. Our results also show that the respiratory complex NQR, an essential enzyme that energizes the membrane and sustains a large variety of homeostatic and infection-related processes (23), is the main molecular target of clofazimine and phenothiazine and Phe analogs in *V*. *cholerae*. Biochemical characterizations and computational analyses indicate that these inhibitors act on the catalytic ubiquinone-binding site, which is a unique structural feature not found in any human protein (24). These results open the door to repurposing clofazimine, as well as other Phes and phenothiazines, to treat *V*. *cholerae* infections, and to produce a novel class of antibiotics to target pathogens that rely on NQR activity.

**Figure 1:**
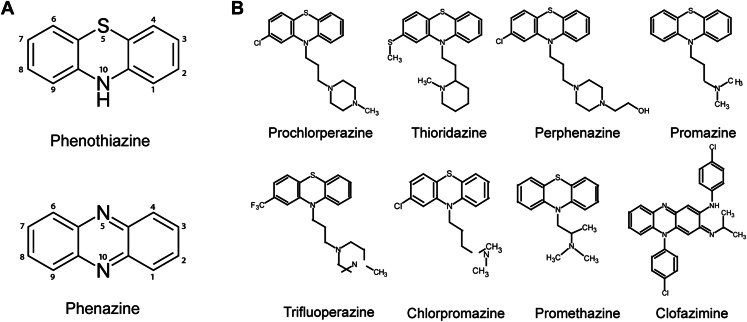
**Chemical structures of phenothiazines and phenazines assessed in this study.** Phenothiazine and phenazine structures (*A*) and derivatives (*B*).

## Results

### Phenothiazine and Phe antibiotic effects on *V*. *cholerae*

**Figure 2:**
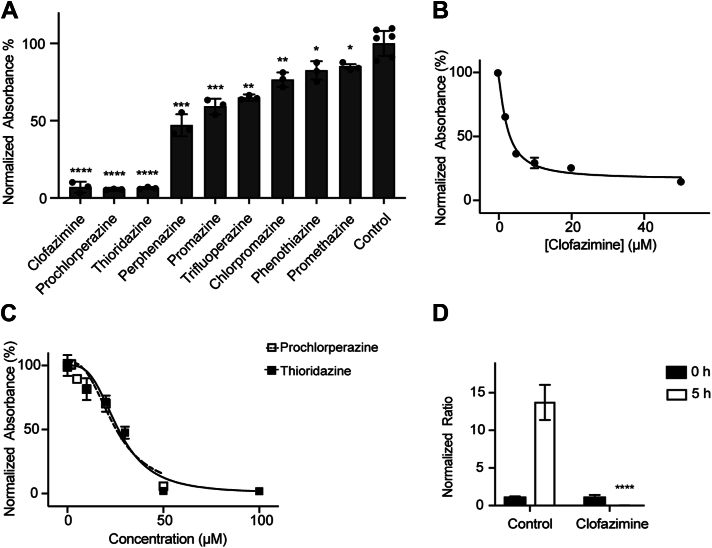
**Effect of phenothiazines and phenazines on *Vibrio cholerae* growth in LB medium**. *A*, *V*. *cholerae* growth at midlogarithmic phase (8 h) in the presence of phenothiazine and phenazine compounds (50 μM). MIC_50_ curves of clofazimine (*B*), prochlorperazine, and thioridazine (*C*) against *V*. *cholerae*. *D*, colony-forming unit (CFU) counts of *V*. *cholerae* in the presence of 10 μM clofazimine or vehicle (control) at the beginning of the experiment (time 0) and after 5 h of growth. Data are expressed as mean ± SD, *n* = 3. *Asterisks* indicate significance from control as determined by *t* test statistical analyses (∗*p* < 0.05, ∗∗*p* < 0.01, ∗∗∗*p* < 0.001, and ∗∗∗∗*p* < 0.0001).

To identify Phe and phenothiazine molecules that could be repurposed as antibiotics against *V*. *cholerae*, we tested the effects of nine FDA-approved and structurally diverse phenothiazines and Phes on cell growth (Fig. 1*B*). As can be observed in Figure 2*A*, clofazimine, prochlorperazine, and thioridazine showed strong inhibitory effects on the growth of *V*. *cholerae* O395, evaluated during the midlogarithmic stage of growth (8 h) in LB medium. Clofazimine has potent antibiotic effects on the growth of *V*. *cholerae*, with an MIC_50_ of 3.4 ± 0.2 μM (Fig. 2*B*). Thioridazine and prochlorperazine displayed moderate antibiotic effects with MIC_50_ values of 27 ± 2 μM and 24 ± 3 μM, respectively (Fig. 2*C*). These experiments were corroborated by carrying out colony-forming unit (CFU) assays (Fig. 2*D*). In addition to lab strain O395, clinical strains *V*. *cholerae* 2010EL-1786 and 2012EL-2176 were included in our evaluation. These strains are highly relevant as they represent two of the main strains involved in the 2010 Haiti humanitarian crisis (25). Both strains display multidrug resistance profiles, with 2012EL-2176 showing additional resistance to beta-lactam antibiotics, such as ampicillin, cephalosporins, and amoxicillin/clavulanic acid. Our data show that *V*. *cholerae* 2010EL-1786 and 2012EL-2176 have minimum inhibitory concentration (MIC) for clofazimine in the same range as the O395 laboratory strain (Table 1), indicating that these strains have not evolved resistance mechanisms for Phes and that clofazimine could be used to treat pandemic strains. Our data show that clofazimine acts primarily as a bacteriostatic agent, with a minimum bactericidal concentration/MIC ratio of 12.8, whereas bacteriocidal agents have a ratio <4 (26). While prochlorperazine and thioridazine have been previously identified as antibiotics against *V*. *cholerae* (27), a systematic analysis has not been carried out and clofazimine remained unidentified as a potential antibiotic.

**Table 1:** MIC of clofazimine for three strains of *V*. *cholerae*

*V*. *cholerae* strain	MIC (μM)
O395	5
2010EL-1786	16
2012EL-2176	16

### Antibiotic and antivirulence properties of clofazimine in human intestinal epithelial cells *in vitro*

**Figure 3:**
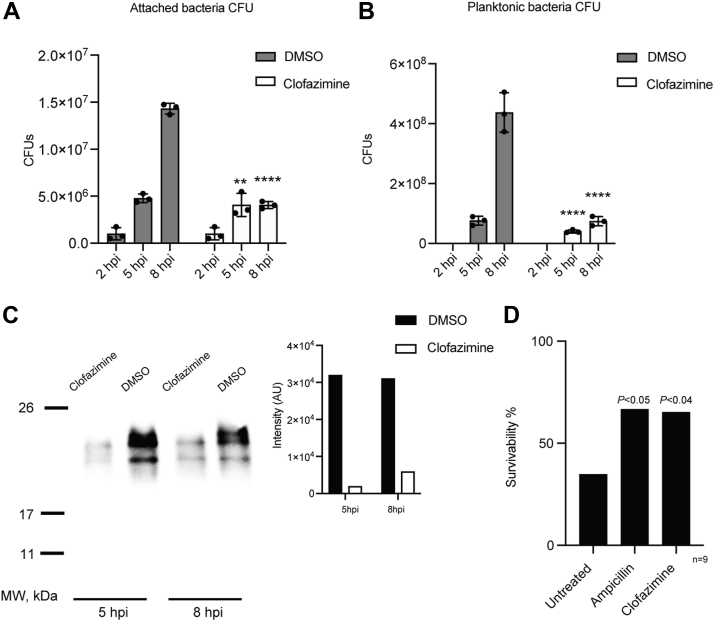
**Effect of clofazimine on *Vibrio cholerae in vitro* infection of T84 human intestinal epithelial cells, cholera toxin production, and on survival of infected mice**. T84 cells were infected with wildtype *V*. *cholerae* at a multiplicity of infection of 10. (*A*) *V*. *cholerae* count (colony-forming unit/ml) postinfection, of attached bacteria and (*B*) planktonic bacteria after 30 μM clofazimine treatment at 2 hpi. Results are presented as mean ± SD, *n* = 3. *Asterisks* indicate significance from vehicle control group (dimethyl sulfoxide) by *t* test analyses (∗∗*p* < 0.01, ∗∗∗∗*p* < 0.0001). *C*, effect of clofazimine treatment at 2 hpi on cholera toxin production at 5 hpi and 8 hpi, analyzed by Western blotting. *Inset*, graphical display of the expression level of cholera toxin. *D*, survivability of mice intragastrically challenged with *V*. *cholerae* cells after treatment with 50 mg/kg ampicillin, 25 mg/kg clofazimine, or vehicle (0.1% Tween-80) (untreated) at 2 and 6 hpi. n = 9. *p* Value is calculated by Chi-squared test.

To further characterize the effects of clofazimine on *V*. *cholerae* infectivity and pathogenicity, an *in vitro* infection model of T84 human intestinal cells was used. T84 cell monolayers were infected with *V*. *cholerae* O395 cells at a multiplicity of infection of 10. After 2 h of incubation, nonadherent bacterial cells were removed by PBS wash. Clofazimine (30 μM) was added at different times, and CFUs were determined for adherent and planktonic cells. Figure 3, *A* and *B* shows that *V*. *cholerae* can effectively colonize T84 cells *in vitro*, reaching densities >1 × 10^8^ in 8 h postinfection (hpi), producing significant numbers of adherent and planktonic cells. Our data also show that the treatment with 30 μM clofazimine significantly reduced the growth of *V*. *cholerae* cells, inhibiting the number of planktonic cells by >90% and attached cells by >75% at 8 hpi. A decrease in the effectiveness of clofazimine compared with LB medium (MIC = 5 μM) was evident, which could be due to clofazimine binding to serum albumin in the growth culture.

Given the crucial role of the cholera toxin in toxicity to mammalian cells, we determined the effects of clofazimine on cholera toxin production in our *in vitro* mammalian cell infection system. Western blot assays were performed against the cholera toxin using the culture media from the infected samples at 5 and 8 hpi. Figure 3*C* shows that our *in vitro* infection system recapitulates the pathogenesis of cholera, as the bacteria can produce the cholera toxin subunit A (23 kDa). Remarkably, the treatment with clofazimine almost completely abolished cholera toxin production (95% and 92% at 5 and 8 hpi, respectively), showing a stronger inhibition than on *V*. *cholerae* growth. Thus, clofazimine appears to have a double action, as an antibiotic, inhibiting growth, and decreasing the production of virulence factors by the surviving population of bacteria.

### Antibiotic properties of clofazimine *in vivo* using a mouse model of intestinal *V*. *cholerae* infection

To determine the properties of clofazimine as an antibiotic, its effects were tested on the survivability of mice infected with *V*. *cholerae* wildtype. The bacteria cultures were grown in AKI media supplemented with 0.3% NaHCO_3_ to induce the expression of the virulence factors (28). Bacterial cells were inoculated into C57BL/CJ 3-week-old female mice by gavage. At 2 and 6 hpi, animals were treated with 50 mg ampicillin/kg, an effective antibiotic against *V*. *cholerae* (29), as positive control, or 25 mg clofazimine/kg, a concentration used in the treatment of tuberculosis (30, 31), or with the vehicle. As observed in Figure 3*D*, <40% of animals subjected to *V*. *cholerae* infection were able to survive the infection challenge. The treatment with ampicillin greatly improved survival, almost twice compared with the untreated group. Remarkably, clofazimine treatment protected infected animals to the same extent as ampicillin (Fig. 3*D*). Our results clearly show that clofazimine could represent an alternative to treat cholera in the clinic. However, further characterizations are required.

### Identification of the molecular target of phenothiazines and Phes in *V*. *cholerae*

**Figure 4:**
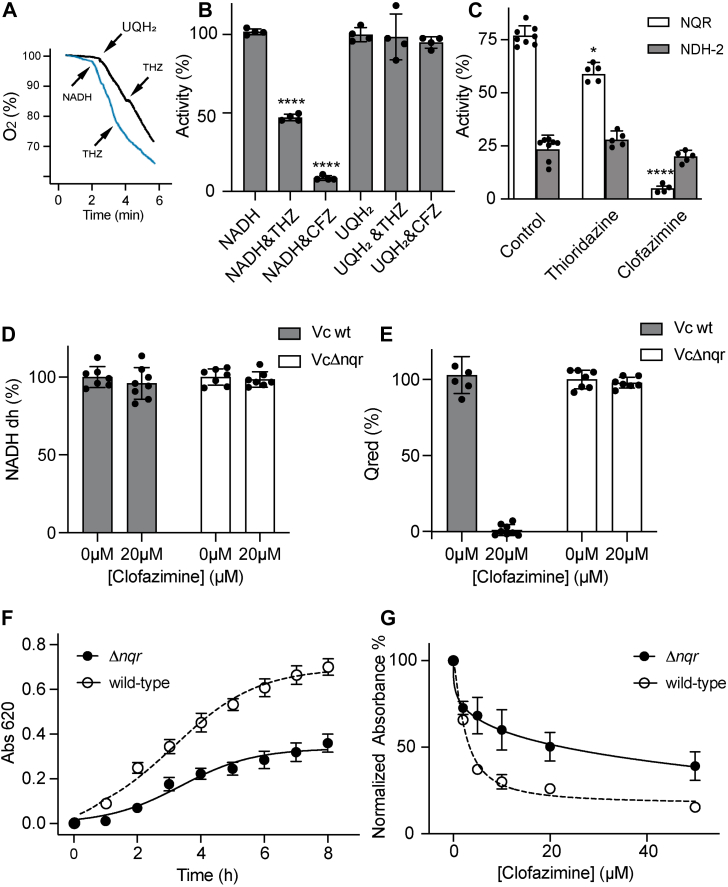
**Effects of thioridazine (THZ) and clofazimine (CFZ) on respiratory activity of *Vibrio cholerae* membranes and on growth of wildtype and Δ*nqr* mutant *V*. *cholerae***. *A*, representative traces of oxygen consumption of *V*. *cholerae* membranes in the presence of 200 μM NADH, 50 μM ubiquinol-1 (UQH_2_), and 50 μM THZ. *B*, respiratory activities of *V*. *cholerae* membranes using 200 μM NADH or 50 μM UQH_2_ with and without 50 μM THZ or CFZ. *C*, NQR and NDH-2 activities in *V*. *cholerae* membranes in the presence of 50 μM THZ or 50 μM CFZ. Data represent the average ± SD, *n* = 5. *D*, NADH dehydrogenase (NADH dh) and (*E*) UQ reductase (Qred) activities in *V*. *cholerae* (Vc wt) and *V*. *cholerae* Δ*nqr* (VcΔnqr) membranes with or without CFZ (20 μM). *F*, comparison of growth curve between wildtype *V*. *cholerae* and Δ*nqr* mutant in LB medium. *G*, MIC_50_ curves of CFZ for wildtype *V*. *cholerae* and Δ*nqr* mutant. Data represent the average ± SD, *n* = 3. *Asterisks* denote significance from NADH (*B*) or from control (*C*) as determined by *t* test analyses (∗*p* < 0.05 and ∗∗∗∗*p* < 0.0001).

As reported here, clofazimine and other phenothiazines have the potential to be used in the treatment of cholera. However, to repurpose and reoptimize these molecules as antibiotics against this and other diseases, it is required to identify their molecular target. Previous reports have shown that thioridazine and clofazimine are potent inhibitors of *Mycobacterium tuberculosis* and *Staphylococcus aureus* respiratory enzymes (32, 33, 34), suggesting that the respiratory chain could also be the target in *V*. *cholerae*. The effects of thioridazine and clofazimine were tested on the NADH-dependent respiratory activity of *V*. *cholerae* membranes. As shown in Figure 4, *A* and *B*, thioridazine and clofazimine (50 μM) are strong inhibitors of the respiratory activity with NADH, whereas the respiratory activity with ubiquinol-1 (UQ-1), which reports the downstream activity by the terminal oxidases, is not sensitive to the inhibitor (Fig. 4, *A* and *B*), indicating that these molecules inhibit NADH dehydrogenases (NADH dhs). *V*. *cholerae* has two enzymes that catalyze the transfer of electrons from NADH to ubiquinone, NQR and NDH-2 (35). NQR is a six-subunit respiratory complex that contains six redox-active cofactors (23, 36). In *V*. *cholerae*, NQR couples electron transfer to the pumping of sodium ions across the plasma membrane (23, 37, 38). On the other hand, NDH-2 is a single-subunit enzyme that uses FAD as its sole cofactor, and its activity is not involved in ion translocation (39). The activities of NQR and NDH-2 can be differentiated by their substrate specificity; while NQR can oxidize both NADH and deamino-NADH (40), NDH-2 is specific for NADH (41). The activity with NADH and deamino-NADH was measured in membranes to estimate the contributions of NQR and NDH-2. As shown in Figure 4*C*, NQR is the main NADH dh in *V*. *cholerae*, representing more than 75% of the respiratory activity, with NDH-2 playing a minor role. Figure 4*C* also shows that NQR is strongly inhibited by clofazimine and thioridazine, whereas NDH-2 is insensitive to these inhibitors at a relatively high concentration (50 μM). To corroborate that NQR is the main target of clofazimine in *V*. *cholerae*, activity measurements were carried out in *V*. *cholerae* membranes of wildtype and a knockout mutant lacking NQR, Δ*nqr* (Fig. 4, *D* and *E*). In contrast with wildtype membranes, which are sensitive to clofazimine (50 μM), the NADH-dependent ubiquinol reductase activity of Δ*nqr* membranes, carried out exclusively by NDH-2, is insensitive to clofazimine. These data allow us to identify, for the first time, that NQR is the molecular target of clofazimine and other phenothiazines and Phes, in *V*. *cholerae* and likely in other bacteria.

To confirm the biochemical data, we analyzed the effects of clofazimine on the growth of wildtype *V*. *cholerae* and Δ*nqr* cells. As shown in Figure 4*F*, the *V*. *cholerae* Δ*nqr* mutant shows a significant defect in growth (>50%) compared with the wildtype strain, which indicates that NQR contributes significantly to the physiology of *V*. *cholerae*, as reported before (42), but it is not absolutely essential. This result explains that clofazimine behaves more as a bacteriostatic rather than a bactericidal agent, as the cells can survive without NQR. In contrast to the wildtype strain, the Δ*nqr* mutant has a reduced susceptibility to clofazimine, with an MIC_50,_ an order of magnitude higher, 23 ± 6 μM, and a resistance of about 40% of the growth to saturating concentrations of this inhibitor (>100 μM) (Fig. 4*G*). Thus, these data further corroborate that NQR is not only the main target of the inhibition in the wildtype strain but also show that there are secondary targets with lower susceptibility to clofazimine in the mutant.

### Phenothiazine and Phe testing on isolated NQR complex

**Figure 5:**
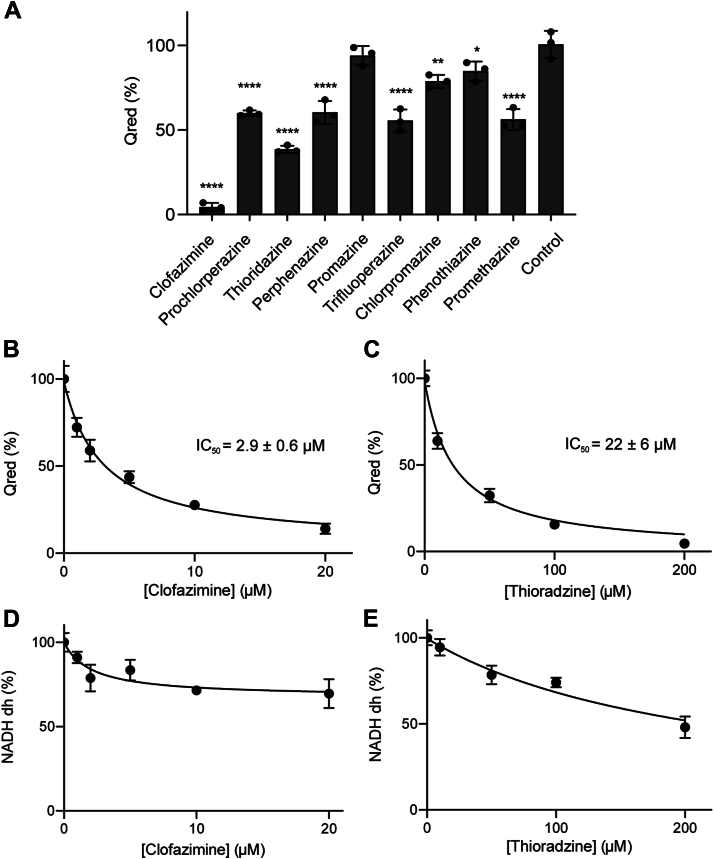
**Effect of clofazimine and thioridazine on the ubiquinone reductase and NADH dehydrogenase activity of NQR**. *A*, NQR ubiquinone reductase activity in the presence of phenothiazine and phenazine compounds (50 μM). The ubiquinone reductase activity was measured in the presence of increasing concentrations of clofazimine (*B*) and thioridazine (*C*). The NADH dehydrogenase activity was measured in the presence of increasing concentrations of clofazimine (*D*) and thioridazine (*E*). Curves of IC_50_ values were determined by nonlinear regression. All data are shown as mean ± SD, *n* = 3.

To understand the action mechanism of these molecules, their inhibitory effects were tested on the purified *V*. *cholerae* NQR complex. In addition to thioridazine and clofazimine, other FDA-approved phenothiazines and Phes were tested (Fig. 5*A*), measuring the ubiquinone reductase (Q_red_) and NADH dh activity of the purified complex, using near-saturating concentrations of the substrates: NADH (250 μM), UQ-1 (50 μM), and NaCl (50 mM). NQR contains three semi-independent functional modules: (1) an NADH dh module, which oxidizes NADH and donates electrons to the cofactors, (2) a ubiquinone reductase module, which catalyzes the physiologically relevant electron transfer from the reduced cofactors to ubiquinone, and (3) an NADH oxidase module, which delivers the electrons from reduced cofactors to oxygen. The NADH oxidase activity is not physiologic, but it is especially active in the absence of ubiquinone or when electron flow is blocked by mutations or in the presence of inhibitors (24). Figure 5*A* shows that all molecules tested produced significant inhibition of the ubiquinone reductase activity, including chlorpromazine, promethazine, perphenazine, and trifluoperazine, which produced a 20% to 40% inhibition of the ubiquinone reductase activity at a concentration of 50 μM. Thioridazine and clofazimine showed inhibitions of 60% and 95%, respectively. Interestingly, prochlorperazine, which showed significant effects on the growth of *V*. *cholerae*, did not produce a comparable inhibition on NQR activity. Figure 5 shows that clofazimine and phenothiazine have strong inhibitory effects on the ubiquinone reductase activity (Fig. 5, *B* and *C*) and negligible effects on the NADH dh activity. This is a typical behavior of compounds that inhibit the ubiquinone site, which does not allow the reduction of this substrate, but do not block NADH oxidation as the electrons are donated to oxygen.

To determine the potency of the inhibitors, the IC_50_ values were calculated, testing the enzyme’s activity at varying concentrations of each compound. Clofazimine has the strongest inhibition on NQR activity, with an IC_50_ = 2.9 ± 0.6 μM (Fig. 5*B*). Thioridazine also showed good inhibitory properties, with an IC_50_ = 22 ± 6 μM (Fig. 5*C*). These two molecules showed low potency inhibition on the NADH dh module (Fig. 5, *D* and *E*), indicating that they act on the ubiquinone-dependent reaction. Previous experiments have shown that clofazimine antibiotic mechanism of action against *M*. *tuberculosis* involves its reduction by the respiratory enzyme NDH-2, which leads to the production of reactive oxygen species (33). In our experiments, clofazimine did not act as a cosubstrate of the reaction and was not reduced by the isolated NQR complex, and it acts as a simple noncovalent but potent inhibitor. The MIC_50_ values obtained in the microbiological analyses are very similar to the IC_50_ measured in the enzymatic assay, further confirming that these two compounds act on NQR as the primary target in the cell.

### Inhibitory mechanism of phenothiazines

**Figure 6:**
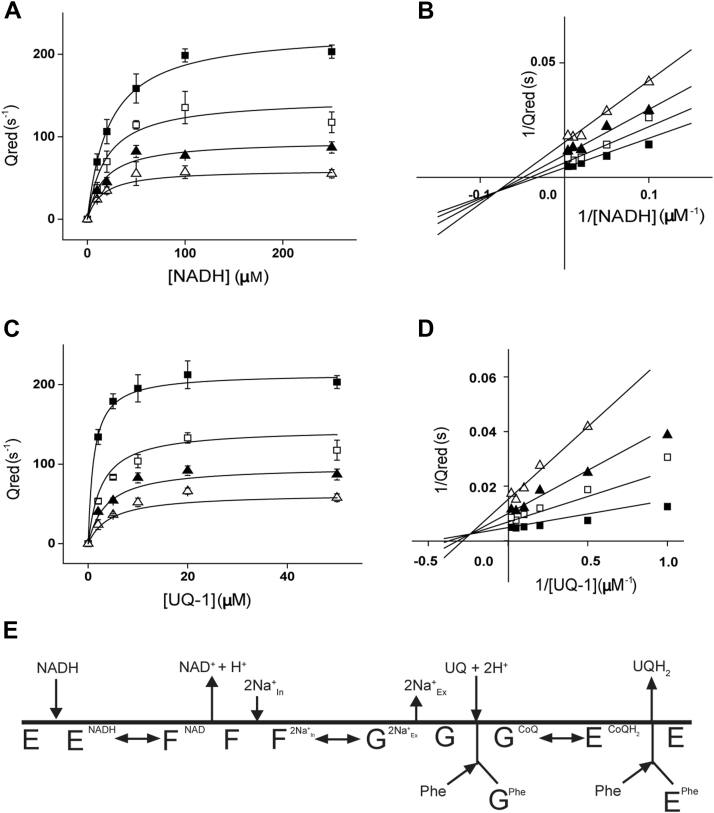
**Inhibition patterns produced by clofazimine**. The concentrations of clofazimine are as follows: 0 μM (◼︎), 2 μM (◻︎), 5 μM (▲), and 10 μM (△). *A*, the concentration of NADH was varied at saturating concentrations of ubiquinol-1 (UQ-1) (50 μM) and NaCl (50 mM). *B*, double-reciprocal plot of data in *A*. *C*, the concentration of UQ-1 was varied at saturating concentrations of NADH (250 μM) and NaCl (50 mM). *D*, double-reciprocal plot of data in *C*. Data were globally fitted to mixed-type inhibition equation. All data are shown as mean ± SD, *n* = 3. *E*, Cleland model of phenothiazine and phenazine inhibition.

To investigate the inhibition mechanism of clofazimine and thioridazine, UQ-1 and NADH titrations were carried out at different concentrations of the two inhibitors. The data were globally fitted to the three basic inhibitory mechanism equations: competitive, uncompetitive, and mixed inhibition. The data with clofazimine (Fig. 6) and thioridazine (Fig. S1) were best fitted to the mixed-type inhibition equation. The double-reciprocal plots show lines intersecting near the *x*-axis, characteristic of this type of inhibition. As shown in Table 2, the two inhibition constants for the competitive and uncompetitive components of the inhibition, *K*_*ic*_ and *K*_*iu*_, respectively, that were obtained in the presence of NADH are similar, indicating that substrate binding does not modify the affinity for the inhibitor, and thus, the sites appear to be physically apart. On the other hand, the two inhibition constants are significantly different when performing the ubiquinone titration. The *K*_*ic*_ with clofazimine is approximately five times lower compared with the *K*_*iu*_, suggesting that the inhibitor site is located close to the ubiquinone site, explaining the strong competitive component of the mixed inhibition. Figure 6*E* shows the Cleland model for the proposed inhibition mechanism of Phes and phenothiazines. As described before by our group, NQR follows a Hexa Uni Ping–Pong mechanism in which the oxidation of NADH, the transport of sodium, and the reduction of ubiquinone are carried out by sequential but independent steps (43). From the inhibition mechanism studies, it can be proposed that clofazimine and thioridazine: (a) have two independent binding sites, such as the NADH and the ubiquinone-binding sites or (b) they are bound to the same site in different conformations or redox states of the enzyme. The ubiquinone-binding sites can be proposed as the main inhibition site, as clofazimine and thioridazine showed strong inhibition of the ubiquinone reduction, with significantly lower effects over the NADH oxidation reaction. Figure 6*E* shows the Cleland model that describes a mechanism in which phenothiazines and Phes interact with the ubiquinone-binding site in two different redox states (E and G), producing the mixed-type behavior. We have proposed that HQNO, a ubiquinone analog with mixed-type inhibition, can be bound to the ubiquinone/ubiquinol-binding site in different redox states of the enzyme, which accounts for the mixed behavior (43). While this model can explain the inhibition by both HQNO and phenothiazines/Phes, the inhibition mechanism could be more complex as NQR contains two ubiquinone-binding sites, one located in the interfaces of subunits B and D (44, 45), and a recently described site located in subunit B (46, 47).

**Table 2:** Inhibition constants

Inhibitor	Substrate	*K*_*ic*_ (μM)	*K*_*iu*_ (μM)
Clofazimine			
	NADH	6.2 ± 2.8	3.5 ± 1.8
	Ubiquinone	0.8 ± 0.3	4 ± 0.8
Thioridazine			
	NADH	35 ± 12	38 ± 14
	Ubiquinone	70 ± 5	37 ± 3

### Phenothiazine inhibition site

**Figure 7:**
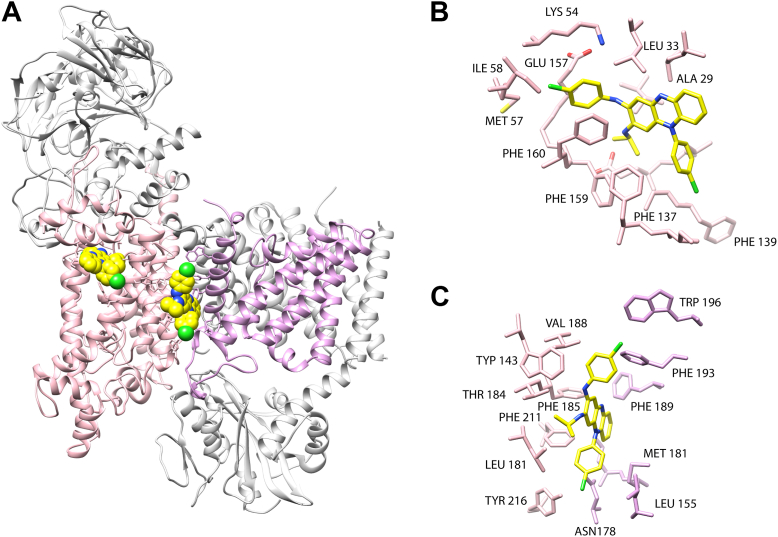
**Molecular docking of clofazimine to *Vibrio cholerae* NQR**. *A*, location of best-scored clofazimine (*yellow*) poses in the reported ubiquinone-binding sites in subunits B (*pink*) and D (*purple*). Close ups of clofazimine in the site found in subunit B linked to the binding of other inhibitors (*B*) and in the catalytic site in the interfaces of subunits B and D (*C*).

Structural analyses were carried out to understand the inhibition mechanism of clofazimine and to locate the inhibitor-binding site. Molecular docking analysis was performed using QuickVina-W, the search grid comprised the complete transmembrane regions of subunits NqrB, NqrD, and NqrE, where the ubiquinone-binding sites are located (45, 47), according to the recently published cryo-EM structures (Protein Data Bank [PDB] ID: 8EVU, 8A1Y, 7XK6, and 7XK7) (46). The results show that clofazimine poses are consistently found around the two described ubiquinone-binding sites (Fig. 7*A*). The best-scored pose is located at a recently described ubiquinone-binding site in subunit B (Fig. 7*B*). The pose is stabilized through π-stacking interaction with residue F160, which has been shown to be involved in ubiquinone binding (PDB ID: 8EVU) (47), and the aminochlorobenzene ring of clofazimine, together with several hydrophobic contacts within the binding pocket. This pocket has been shown to harbor ubiquinone analogs that act as inhibitors, such as HQNO (PDB ID: 8A1Y), aurachin D-42 (PDB ID: 7XK6), and korormicin A (PDB ID: 7XK7) (47). In addition, seven of the 10 best-scored poses were located at the interface of subunits NqrB and NqrD (Fig. 7*C*), where a second, catalytically active ubiquinone-binding site is located (45). In this site, the inhibitors make close contacts with residues F185 and F211, which as shown by our group directly participate in ubiquinone binding (45).

To identify the binding site of clofazimine, inhibition titrations were carried out in the mutants of the two ubiquinone-binding sites: B-F160A and B-211A. As shown in Table 3, the IC_50_ value for clofazimine in the B-F160A mutant is very similar compared with the wildtype enzyme. On the other hand, the mutant B-F211A shows an increase in the IC_50_ of nearly three times, indicating that this residue participates in clofazimine binding in the site. Our results strongly suggest that clofazimine interacts with the catalytically active ubiquinone-binding site, located at the interface of subunits B and D. Identification of this putative site is critical to guide future optimization of clofazimine and its analogs tailored to the unique features of NQR, potentially advancing the development of more effective antibiotics against cholerae.

**Table 3:** IC_50_ values for clofazimine of wildtype *V*. *cholerae* NQR and ubiquinone-binding site mutants

Mutants	IC_50_ (μM)
Wildtype NQR	2.9 ± 0.6
NqrB-F160A	1.4 ± 0.5
NqrB-F211A	8.6 ± 0.3

## Discussion

### Repurposing of clofazimine to treat cholera

The increase in antimicrobial resistance has become a serious global health problem. Due to the large investment in time and effort to develop new pharmaceuticals, the pipeline to develop new antibiotics has dried up and considerable efforts have been directed at repurposing existing drugs as antibacterials. In this work, screening of FDA-approved phenothiazines and Phes led to the identification of clofazimine, a riminophenazine, and thioridazine, a phenothiazine, as potential alternative antibiotics to treat cholera. Numerous studies have shown that in addition to their antipsychotic impact, phenothiazines can be utilized as antibiotics (48, 49, 50, 51). For instance, chlorpromazine has shown strong antimicrobial properties against Mycobacteria, Staphylococci, Enterococci, and *Vibrio* spp. (17, 52), and as shown in this work, it also has antimicrobial properties against *V*. *cholerae*. Phenothiazines have also shown antibiotic activity against *M*. *tuberculosis* (32). While their mechanism of action has remained obscure, phenothiazines have been described as inhibitors of quorum sensing in *Vibrio harveyi* (53). Moreover, phenothiazines have been associated with the inhibition of antibiotic resistance mechanisms. In particular, chlorpromazine appears to reverse antibiotic resistance (54), and phenothiazines in general seem to indirectly inhibit efflux pumps in different bacterial species (52, 55, 56), which may potentiate antimicrobial activity by increasing the intracellular concentration of antibiotics (57).

Although clofazimine is considered an orphan drug, it is an established part for the treatment of multidrug-resistant leprosy (58). More recently, clofazimine has been used to treat multidrug-resistant tuberculosis, showing positive outcomes in a significant number of patients (22, 59). Clofazimine has also been identified as a potential therapeutic against *Cryptosporidium parvum*, showing potent effects on *in vitro* proliferation, and a significant reduction of oocyte shedding in a murine infection model (60). Clofazimine has been described as a safe and well-tolerated drug that has minor side effects (61), which together with new technologies to deliver lipophilic drugs, make clofazimine a suitable candidate as antibiotic (62). In this report, we are showing that at concentrations of 10 to 30 μM, clofazimine inhibits the growth of *V*. *cholerae* in both culture and in an *in vitro* infection model of intestinal cells. This concentration is well below the clinical range, 100 mg per day for adults (63). Considering that the stomach’s volume is approximately 1.5 l (64), we estimate that the concentration of clofazimine that can reach the proximal section of the small intestine is around 150 μM, several times higher than the concentration used here. While the concentration of clofazimine is relatively high in the gastrointestinal tract, the concentration in plasma is in the submicromolar range (65), which makes it a safe alternative to treat gastrointestinal pathogens like *V*. *cholerae*. The extrapolation of *in vitro* data to *in vivo* behavior is not straightforward because of variability in absorption after oral administration, produced in part by individual food intake and content in the gastrointestinal tract (65). However, our data show that the same single oral dose of clofazimine (25 mg/kg) used here is used in *M*. *tuberculosis* infection model. In that study, the peak serum concentration after single dose was 0.43 μg/ml (31), which is in the rage found for human patients when administered with a single dose of oral clofazimine of 100 mg or 300 mg and sera concentration of 0.7 to 1 μg/ml (61). Thus, the concentrations used here are clinically relevant. In addition to halting bacterial cell growth, clofazimine has antivirulence properties as it almost completely decreases the production of the cholera toxin by the pathogen, through NQR inhibition (see later), which makes it an even more attractive compound. Our results show that the *in vitro* effects are recapitulated *in vivo* in a mice model of *V*. *cholerae* infection. According to our data, clofazimine is as effective as ampicillin to prevent mice death induced by the pathogen. Moreover, clofazimine is effective in the same range against laboratory and pandemic-producing *V*. *cholerae* strains, including those involved in the 2010 Haiti humanitarian crisis. The results presented here open a window to repurpose this relatively inexpensive and safe orphan drug to potentially treat multidrug-resistant *V*. *cholerae* infections.

### NQR as the target of phenothiazines and Phes

Although Phes and phenothiazines have shown promising antibiotic properties, their molecular targets have remained largely unknown. These molecules have been reported as inhibitors of efflux pumps (66, 67) and as agents that promote the elimination of plasmids carrying drug-resistance genes from pathogens (67, 68). In this study, we are demonstrating that the cellular target of these antibiotics in *V*. *cholerae*, and likely other bacteria, is the NQR complex, which plays essential roles in different microorganisms (69). Interestingly, clofazimine has activity against Mycobacteria by inhibiting menaquinone-using enzymes, such as NDH-2 (70), which carries a similar function compared with NQR. NQR serves as the entry point of electrons into the respiratory chain as well as the primary sodium pump in hundreds of pathogenic bacteria (23, 38, 43, 69). NQR activity generates a sodium gradient that facilitates essential physiologic functions, including flagella rotation, ATP synthesis, nutrient transport, and virulence factor secretion (69, 71, 72, 73). In addition, the electrochemical gradient is used directly by efflux pumps to eliminate antibiotics (74), which appears to be a major factor involved in the prevalence of this enzyme in multidrug-resistant microorganisms (75). NQR inhibition by clofazimine and phenothiazines can explain the observed reversal of antibiotic resistance, as antibiotic efflux would be indirectly inhibited by membrane de-energization prompted by NQR inhibition. Moreover, the decrease in toxin production can also be explained by NQR inhibition, as *V*. *cholerae* Δ*nqr* mutant is almost completely avirulent. Deletion of NQR significantly reduces the secretion of cholerae toxin, up to eightfold depending on strain, to reduce bacterial motility in liquid culture media, as well as attenuated toxin-corregulated pilus–related phenotypes (76). Also, it was observed that deletion of NQR reduced colonization in an infant mouse model (77).

Previous work with *Chlamydia trachomatis* have proposed that NQR can be targeted using novel antibiotics (78). However, this report represents the first case in a drug that specifically inhibits that NQR is used as an antibiotic. The data presented here show that NQR is an optimal cellular target in *V*. *cholerae* since (1) it is expressed exclusively in bacteria and lacks a mammalian mitochondrial homolog, (2) carries essential functions as a respiratory enzyme and as the main ion pump in the cell, (3) regulates both virulence and antibiotic resistance processes, and (4) it is an evolutionary-divergent enzyme that carries unique structural motifs not found in any human enzyme (78), including the catalytically active ubiquinone-binding site, which is targeted by clofazimine, as shown in this report. The findings of this report pave the way for the development of a novel class of antibiotics that target NQR as an essential enzyme in pathogenic bacteria.

## Experimental procedures

### Cloning, protein expression, and purification

The wildtype *nqr* operon from *V*. *cholerae* was cloned into the pBAD/HisB as described previously (43). Single mutations in NqrB, F160A, and F211A were produced by site-directed mutagenesis using the pBAD/HisB-*nqr* construct as a template as described before (79). The pBAD/HisB-*nqr* constructs were transformed into the *V*. *cholerae* O395 strain with a deleted genomic *nqr* operon (Δ*nqr*) for subsequent protein expression. The transformed cells carrying the expression plasmid were grown in LB media containing 100 μg/ml ampicillin and 50 μg/ml streptomycin at 37 °C with agitation (250 rpm). The genetic expression of NQR was induced with 0.05% arabinose. The induced cells were harvested and washed by centrifugation and then lysed *via* high-pressure homogenizer (Emulsiflex-C5; Avestin) in the presence of PMSF and DNase I. Cytoplasmic membranes were obtained by ultracentrifugation. The membranes were solubilized in buffer containing 0.05% n-dodecyl-β-d-maltoside (DM), 5 mM imidazole, 50 mM Na_2_HPO_4_, 300 mM NaCl, 5% glycerol, pH 8.0. The NQR complex was purified by two steps of chromatographic purification: Ni–NTA affinity chromatography and DEAE ion-exchange chromatography. The composition of enzyme subunits and protein purity (>92%) were confirmed by urea (30%) and SDS-PAGE 15% acrylamide gels.

### *V*. *cholerae* membrane preparation

*V*. *cholerae* O395 cells were harvested at the early stationary phase and then washed by centrifugation using KHE buffer (150 mM KCl, 20 mM Hepes, 1 mM EDTA, pH 7.5) (80). The cells were lysed through Emulsiflex-C5 homogenizer with PMSF and DNase I at 16,000 psi. Cell debris was eliminated by differential centrifugation. The membranes were collected *via* ultracentrifugation and then were washed and resuspended in KHE buffer and stored at −80 °C. Membrane protein concentration was measured using Pierce BCA Protein Assay Kit (Thermo).

### Enzymatic activity and IC_50_ measurements

Enzyme activity was measured following the ubiquinone reduction at 282 nm and NADH oxidation at 340 nm (43, 79, 81), with the substrates UQ-1 and K_2_-NADH. The preliminary screening reactions were performed in TEG buffer (50 mM NaCl, 50 mM Tris, 1 mM EDTA, 5% glycerol, 0.05% DM, pH 8.0) containing 2.5 nM enzyme, 250 μM K_2_-NADH, 50 μM UQ-1, and 50 μM phenothiazine or Phe compounds. The potential compounds were selected with a percentage of inhibition over 40%. The IC_50_ assay was started by mixing 2.5 nM of enzyme, saturating concentrations of UQ-1 (50 μM) and K_2_-NADH (250 μM) with different concentrations of thioridazine (0–100 μM) or clofazimine (0–10 μM). UV–visible spectra were recorded on a Cary 8454 UV–visible diode array system (Agilent Technologies). Initial rates were obtained to represent enzyme activity. All experiments were performed in triplicate, and the experimental data were analyzed *via* GraphPad Prism (version 9.2.0; GraphPad Software, Inc).

### Inhibition assays

UQ-1 and NADH titration experiments were performed to characterize the inhibition mechanism of thioridazine and clofazimine against NQR. Initial rates were measured through UQ-1 (1, 2, 5, 10, 20, and 50 μM) and NADH (10, 20, 50, 100, and 250 μM) titrations at different concentrations of thioridazine (0, 10, 50, and 100 μM) or clofazimine (0, 2, 5, and 10 μM). The rates calculated were then fitted to different types of inhibition models: competitive, uncompetitive, and mixed inhibitions. The parameters were calculated from a double-reciprocal plot. All experiments were performed in triplicate, and the experimental data were analyzed using OriginLab Origin 8.1 (OriginLab Corporation).

### Oximetry

A Clark-type electrode YSI 5300 fitted to a 1.6 ml custom glass chamber was used to measure the oxygen consumption rate of *V*. *cholerae* membrane NADH dhs with thioridazine. The reaction took place in an electrolyte solution (150 mM KCl, 50 mM NaCl, 50 mM Hepes, and 1 mM EDTA, pH 7.5), which provides sufficient Na^+^ for the NADH dhs electron transport at 37 °C. NADH dh activity was tested using 200 μM NADH. Ubiquinol oxidase activity was determined using 50 μM UQ-1 in the presence of 500 μM DTT. Thioridazine (50 μM) inhibition was measured *via* 30 s incubation with *V*. *cholerae* membranes.

### NADH dh activity and inhibition measurements

*V*. *cholerae* membranes were used to measure the NADH dh activity. DM (0.05%) was added into the membrane sample and then was used to measure the dehydrogenase activity at 340 and 282 nm in KHE buffer with substrates. KCN (10 mM) was added to block the electron transport to molecular oxygen through terminal oxidases. The reaction mixture included 20 ng/μl membrane, 50 μM UQ-1, and 50 μM thioridazine. The assay started by immediately mixing the reaction mixture with 250 μM NADH or 250 μM deamino-NADH. Absorbance changes were monitored with a SynergyHTX multimode plate reader (BioTek). All experiments were performed in quintuplicate.

### Antimicrobial assays

The growth curve of wildtype *V*. *cholerae* O395 was obtained in the presence of different concentrations of thioridazine and clofazimine. The efficacy of the compounds is presented as MIC_50,_ and the growth curve measurement was based on absorbance value. The *V*. *cholerae* cells were cultured in LB medium. The final concentrations of thioridazine and clofazimine are 10, 20, 30, 50, and 100 μM, and 1, 2, 3, 4, 5, 6, 8, 10, and 20 μM, respectively. All assay cultures were incubated at 37 °C with constant agitation (250 rpm). Dimethyl sulfoxide as the solvent of clofazimine was added into the LB media as control. Absorbance was measured at a wavelength of 620 nm, and the mean of three separate experiments was recorded. To conduct growth curve analyses of wildtype and mutants of *V*. *cholerae*, overnight cultures of wildtype *V*. *cholerae* and the Δ*nqr* mutant were inoculated in sterile LB broth. Each culture (200 μl) was incubated in a sterile, clear, flat-bottomed 96-well microplate. At least eight technical replicates were included per experiment. The mean of three separate experiments was recorded. A BioTek 800 TS microplate reader was used to measure absorbance at 620 nm at 1-h intervals in microplates maintained at 37 °C with regular shaking over an 8-h time course. MIC of wildtype and Δ*nqr* mutant *V*. *cholerae* was performed in 96-well microplates. Cultures were inoculated and incubated with different concentrations of clofazimine (0, 2, 5, 10, 20, and 50 μM) at 37 °C for 8 h. At the end of the incubation period, microplates were read using BioTek 800 TS microplate reader at 620 nm.

### T84 cell infection assay

T84 human intestinal carcinoma cells (American Type Culture Collection CCL-248) were cultured in Dulbecco′s modified Eagle′s medium/Nutrient Mixture F-12 Ham supplemented with 5% fetal bovine serum and penicillin–streptomycin (100 U/ml and 0.1 mg/ml, respectively). After trypsinization with 0.25% trypsin–EDTA, cells were seeded at a density of 2.1 × 10^5^ cells/cm^2^ into 48 wells and incubated at 37 °C with 5% CO_2_ in a humidified atmosphere. *V*. *cholerae* was grown in LB at 37°C and 250 rpm agitation to reach an approximate 1 × 10^9^ CFU/ml. The bacteria cells were then centrifuged at 16,000*g* for 5 min and washed with PBS. Before infection, T84 cells were washed with PBS, and media were replaced with Dulbecco′s modified Eagle′s medium F12 without antibiotics. The cultures were infected with *V*. *cholerae* cells at a multiplicity of infection of 10. The infected monolayers were incubated for 2 h to allow the attachment of *V*. *cholerae* cells to the T84 cells. At 2 hpi, nonattached planktonic bacteria were removed through PBS washes. Freshly dissolved clofazimine (30 μM) was added to the infected cells and incubated until 5 and 8 hpi. Supernatants and the first PBS wash were collected for planktonic CFU determination. Washed infected monolayers were lysed with Triton 0.1% and by mechanical scrapping, and the adherent bacteria were collected and processed for CFU determination in LB agar plates.

### Mouse model of *V*. *cholerae* infection

*V*. *cholerae* infection in mice was carried out using a published protocol with minor modifications (82). Wildtype *V*. *cholerae* was grown in AKI medium containing 0.3% NaHCO_3_ at 37 °C under agitation (250 rpm). Bacterial cells were harvested at midlog phase and were washed with PBS by centrifugation (3000 rpm for 30 min). Mice inoculations were performed according to Illinois Institute of Technology Institutional Animal Care and Use Committee–approved protocols. C57BL/CJ female mice of 3 weeks old (The Jackson Laboratory) were anesthetized with 60 mg ketamine/kg of body weight and 12.5 mg of xylazine/kg. Animals were given 50 μl of 8.5% NaHCO_3_ intragastrically followed by 1.5 × 10^7^ *V*. *cholerae* CFUs. Animals were maintained under *ad libitum* feeding conditions with access to sterile water. At 2 and 6 hpi, animals were treated with 50 mg ampicillin/kg, 25 mg clofazimine/kg (dissolved in 0.1% Tween-80), or vehicle. Survivability of the animals was recorded, and the experiment was ended at 24 hpi.

### Western blotting

The cholera toxin content of T84-infected cultures was determined by Western blot analysis. Cell culture supernatants were collected at 5 and 8 hpi and processed for bacterial cell lysis. Samples were treated with 0.1% SDS and boiled for 10 min. Protein per sample (50 μg) were resolved by SDS-18% PAGE and electroblotted onto a polyvinylidene fluoride transfer membrane. Blots were probed with anti–cholera toxin antibodies (1:10,000 dilution) (Sigma–Aldrich). Antibodies were detected with the SuperSignal West Femto Maximum Sensitivity Substrate (Thermo). Signals were detected with a ChemiDoc Imager system with Image Lab Touch Software (Bio-Rad). All detected signals were unsaturated and in the linear range of detection. The intensity of the bands was analyzed densitometrically using ImageJ (83).

### Molecular docking

Clofazimine was docked into the membrane-bound B, E, and D subunits of NQR (PDB ID: 8EVU) with AutoDock Vina 1.1.2 (84), using the SMILES string from the ZINC database (85) (accession number: ZINC17953024). Clofazimine structure was built and minimized using UCSF Chimera 1.14 (86). Polar hydrogens and Gasteiger charges were added to the homology model using Dock Prep in Chimera.

## Data availability

All data used to make the figures are contained within this article.

## Supporting information

This article contains supporting information.

## Conflict of interest

The content is solely the responsibility of the authors and does not necessarily represent the official views of the National Institutes of Health.
