# Supplementary material for: Repurposing clofazimine as an antibiotic to treat cholera: Identification of cellular and structural targets
Source: J Biol Chem. 2025 Jul 4;301(8):110458. doi: 10.1016/j.jbc.2025.110458 (PMC12336824; doi:10.1016/j.jbc.2025.110458)
Supplement: Supplementary Material [file mmc1.pdf]

Supporting Information

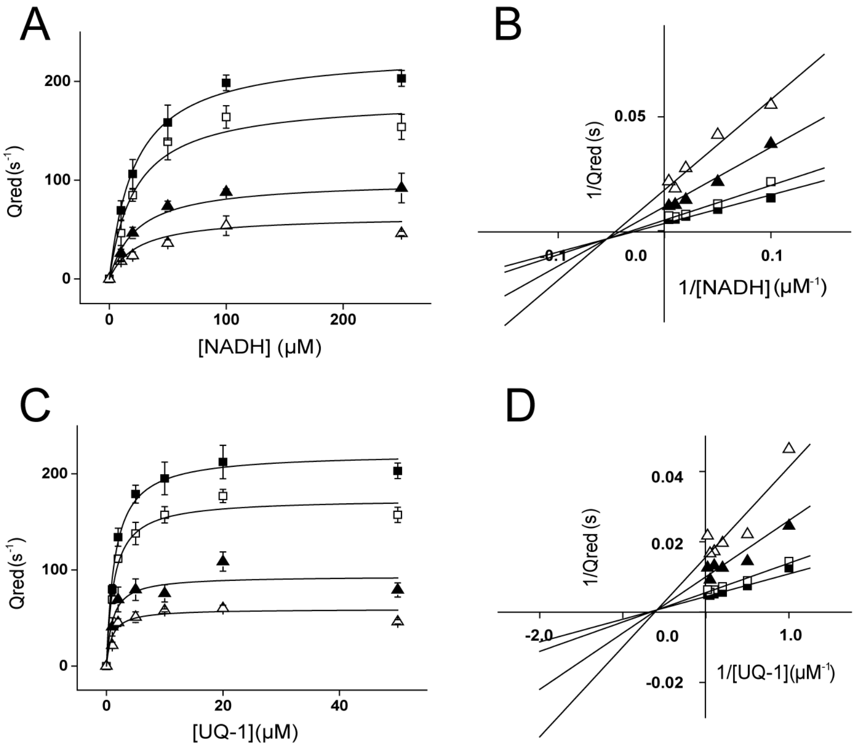

Fig. S1. Inhibition patterns produced by thioridazine. The concentrations of thioridazine are as follows: 0 µM (○), 10 µM (●), 50 µM (△), and 100 µM (▲). (*A*) Concentration of NADH was varied at saturating concentrations of UQ-1 (50 µM) and NaCl (50 mM). The double-reciprocal plot of this data set is shown in (*B*). (*C*) Concentration of UQ-1 was varied at saturating concentrations of NADH (250 µM) and NaCl (50 mM). The double-reciprocal plot of this data set is shown in (*D*). Data were globally fitted to mixed type inhibition equation. All data are shown as mean ± SD, *n* = 3.
